# Cascaded Thermodynamic and Environmental Analyses of Energy Generation Modalities of a High-Performance Building Based on Real-Time Measurements

**DOI:** 10.3390/e22040445

**Published:** 2020-04-14

**Authors:** Raaid Rashad Jassem Al Doury, Saadet Ozkan, M. Pinar Mengüç

**Affiliations:** 1 Center for Energy, Environment and Economy (CEEE/EÇEM), Ozyegin University, Cekmekoy, Istanbul 34794, Turkey; raaidaldoury@tu.edu.iq; 2 Department of Mechanical Engineering, Tikrit University, Salah Al-Deen 34011, Iraq; 3 Energy Distribution Center, Ozyegin University, Cekmekoy, Istanbul 34794, Turkey; saadet.ozkan@ozyegin.edu.tr

**Keywords:** energy efficiency, dynamic energy analysis, exergoeconomic analysis, sustainability index, high-performance buildings, tri-generation system, PV panels, ground-air heat exchanger system

## Abstract

This study presents cascaded thermodynamic and environmental analyses of a high-performance academic building. Five different energy efficiency measures and operation scenarios are evaluated based on the actual measurements starting from the initial design concept. The study is to emphasize that by performing dynamical energy, exergy, exergoeconomic, and environmental analyses with increasing complexity, a better picture of building performance indicators can be obtained for both the building owners and users, helping them to decide on different investment strategies. As the first improvement, the original design is modified by the addition of a ground-air heat exchanger for pre-conditioning the incoming air to heat the ground floors. The installation of roof-top PV panels to use solar energy is considered as the third case, and the use of a trigeneration system as an energy source instead of traditional boiler systems is considered as the fourth case. The last case is the integration of all these three alternative energy modalities for the building. It is determined that the use of a trigeneration system provides a better outcome than the other scenarios for decreased energy demand, for cost reduction, and for the improved exergy efficiency and sustainability index values relative to the original baseline design scenario. Yet, an integrated approach combining all these energy generation modalities provide the best return of investment.

## 1. Introduction

Buildings contribute to about 35–40% to the energy used in almost all climate zones. Possible reductions of energy consumption in buildings are some of the most important strategic goals to combat the climate change [1,2,3]. This goal can be achieved by following an integrated approach in the design and operation of buildings by architects and engineers. However, such studies require a cascaded thermodynamic, environmental and economic analysis of buildings, and the development of a comprehensive sustainable energy roadmap, involving multitudes of alternative energy technologies. These complicated problems cannot be understood using simply the conservation of energy principles. Instead, analyses using both the first and the second laws of thermodynamics need to be considered. This requires combining exergy and energy analyses towards the goal of sensible improvements in building performances [3,4,5,6].

In addition to reducing energy consumption, all possible improvements should be assessed based on their long-term economic benefits and their direct impact on occupancy comfort parameters. Only then will stakeholders be able to choose the most sustainable alternative energy technologies for new buildings or decide how to proceed with the renovation of existing ones. In this paper, we outline a cascaded thermo-economic analysis based on exergoeconomic, environmental impact and sustainability models. The present approach combines the cost of input energy, capital investment, and exergy values of input and output streams of each component in a building, and uses real data obtained at different phases of the building operation. We systematically analyze four different scenarios beyond the original design conceptualized by the architects of the building. Three of these four scenarios are based on major energy generation modalities chosen by the University and studied in detail by the researchers at the Center for Energy, Environment and Economy (CEEE/EÇEM). They include a ground heat pipe system, roof-top PV panels and the use of a trigeneration system. The last scenario corresponds to the combination all of these systems.

There are several studies available in the literature which discuss similar problems based on thermodynamic principles [7,8,9,10,11,12,13,14]. Schmidt and his co-workers emphasized that the energy approach is not sufficient to study complex problems and must exergy modeling should be included [8,9,10]. Tsatsaronis also presented an analysis to provide the designers an opportunity to optimize the components and processes to achieve a cost-effective solutions [12]. His method allows researchers to connect many aspects that are related to inefficiencies such as exergoeconomic and environmental concerns. A review of low exergy heating and cooling systems and applications for sustainable buildings and regions was conducted by Hepbasli [15]. Using exergy analysis, Balta et al. estimated the performance of a building for four options of heating applications based on renewable and nonrenewable energy sources, including a heat pump, condensing boiler, conventional boiler, and a solar collector [16].

As part of improving an existing building, Jansen et al. [17] presented the exergy performance of a social dwelling of a multi-family building in Bilbao (Spain). They proposed and investigated the improved energy concepts according to smart exergy use. In another study, Yucer and Hepbasli [18]. estimated the exergoeconomic and enviroeconomic performance by applying specific exergy costing (SPECO) method to a heating system of a building. The exergetic cost effectiveness (ECE) was used to determine the most effective components that should be improved. In order to achieving the same goal, Caliskan [19] applied the lowex analysis on a building with a ground source water heat pump that was used to heat a building. The building was heated using floor heating. A similar case was also studied by Wei and Zmeureanu [20], however, they only considered two types of variable air volume (VRV) systems instead of a single one used extensively.

Sevilgen and Sancar [21] developed a model to estimate the best capacity of a trigeneration system. Their model was based on adding the trigeneration system to existing heating system and creating an optimized operation strategy. Acikkalp et al. [22] analyzed buildings and their energy production sub-components exergetically and exergoeconomically in order to determine potential improvements to the entire system. They also suggested a number of modifications for developing an economic operation of the building. A combined cooling, heating, and power (CCHP) system was analyzed and compared with the original design, as well as with three improvements, including addition of a gas turbine, a heat recovery steam generator, and a double-effect absorption chiller [23]. At the same time, the packed bed storage system factor effects and its economic aspects were investigated in [24]. Kilkis and Kilkis [25] developed new exergy metrics for the performance analysis and rating of nZEXAP airports, based on an energy, economy, and environment nexus. This optimization problem has three primary design variables, namely the ratio of the optimum cogeneration engine capacity to the peak power load, the split of the generated power supply between the airport and the ground source heat pumps, and the natural gas to biogas mixing ratio.

Martinopoulos et al. [26] considered a typical nine-story office building in the center of Thessaloniki (Greece) and assessed a number of different building-integrated retrofitting measures. Solar energy and shading options were considered in the study using the EnergyPlus software. They concluded that the photovoltaic system can cover 65% of the total annual energy demand whereas the shading options reduce the energy load by about 33%. They have further investigated the design decisions, construction and operational solutions, and thermal behavior of buildings and explored the parameters such as the best orientation of the building, the effect of internal insulation, and the alternating heating system on the annual energy demand reduction [27]. On the other hand, the integration of Solar Water Heating (SWH) systems into High Energy Performance (HEP) housing in Algeria was investigated energetically and economically by Sami et al. [28]. The results revealed very promising high values of the solar fraction in almost all the studied regions. They commented that an adequate funding policy would permit to establish a good balance between system performance and system design resulting in a higher competitiveness of solar energy against conventional energy. Christodoulos and Georgios [29] optimized a building-integrated solar thermal system with seasonal storage with the use of TRNSYS modeling software in order to evaluate different integration options of the solar collector array.

The objective of the present article was to study the performance of an existing high-performance building with real data obtained during different phases of operation and determine the required energy generation and utilization strategies for its long term financial and sustainable operation. For this purpose, we have developed a cascaded methodology which account for energy demand, exergy demand, energy losses, exergy destructions and losses, energy cost, CO_2_ emission, as well as the cost pollutants. We applied the methodology to five different cases mentioned above. The selected scenarios were not arbitrary, but chosen based on the improvements of an actual building constructed with contributions from a EU-FP7 grant and based on the stakeholders’ (the university and the architectural firm) decisions. The timing of the building construction, financing of the improvements, the EU-FP7 grant to CEEE allowed a very opportune time to conduct this study based on actual construction and operation data. If it were a theoretical study, we could have considered a number of other energy efficiency measures; however, here we limit ourselves to three major improvements mentioned above and detailed next.

In this study, we analyze the energy and exergy flows from the beginning (energy source) to the end (building envelope) based on the real data obtained for the actual building. As the first case, we consider the original design of the building and its heating system as designed by architects and engineers (without any suggestions for improvements by the CEEE/EÇEM researchers). Then, we study four additional scenarios to determine the possible improvements of sustainable energy flow in the building. The second case involves the use of Ground Air Heat Exchanger (GAHX) that corresponds to almost 5% of heating load. The third case is the use of roof-top solar PV panels; we note that about 75% of the roof area of the building was used for solar electricity production. The fourth case is the use of a tri-generation system as an energy source instead of the current traditional boiler systems. The tri-generator actually provides the energy for the entire campus, the School of Languages (SCOLA) being one of the buildings considered in this study. Finally, we consider as the fifth case all three alternative energy additions together. Energy, exergy and exergoeconomic analyses are applied dynamically during the entire heating season to all these cases in order to make a detailed comparison between all these strategies. The energy and exergy flows, energy and exergy losses, CO_2_ emission amount, and the energy costs of all of these cases are determined. The exergy efficiency and the sustainability index for each case are calculated. For this purpose, a computational tool was developed by using EES software that can allow us to estimate the benefits of any potential improvements of the building based on dynamic analysis. Finally, a number of improvements are suggested to reduce the adverse effects of the energy use of the building to the environment. In addition, we discuss how to improve the sustainability index of the building based on integrated engineering-architecture principles. The comparisons of sustainability index for different operation cases allow sustainability analysis of high-performance buildings based on fundamental thermodynamic principles.

All analyses have been performed dynamically in order to have higher accuracy in the results obtained as shown in a previous study [30]. The heating loads, energy flows, exergy flows, energy demands, exergy demands, energy losses, exergy losses, CO_2_ amount, and energy demand costs of each case are quantified for the entire heating season. The cooling load analysis might be investigated in a forthcoming work.

## 2. Mathematical Analysis

For all calculations, we used a relatively straightforward EES software based on the guidance of IEA ECBCS Annex 49 (2011) [6]. The governing equations used are given below and reported in detail by Schmidt 2019 [10].

Heat transfer through the building envelop can be determined from [10,31]:

(1) $${\dot{Q}}_{trans}=\sum \left(U_{k}\cdot A_{k}\right)\cdot \left(T_{R}-T_{o}\right)$$

whereas the ventilation losses can be obtained using [18].

(2) $${\dot{Q}}_{v}=\rho_{Air}\cdot V_{buil.}\cdot n_{v}\cdot \left(1-\eta_{Hrecov}\right)\left(h_{R}-h_{o}\right)$$

Please note that the definitions of each symbol are given in the Nomenclature, and therefore are not repeated in the text. For the initial calculations, the solar and internal gains were not considered, although for later calculations they were included. Heating energy and exergy of the building are calculated from:

(3) $$\phi_{h}=\left({\dot{Q}}_{trans}+{\dot{Q}}_{v}\right)-\left({\dot{Q}}_{l}+{\dot{Q}}_{s}+{\dot{Q}}_{occ}+{\dot{Q}}_{app}\right)$$

where the thermodynamic exergy (maximum available work) can be determined from:

(4) $$\psi_{h}=\phi_{h}\cdot \left(1-\frac{T_{0}}{T_{R}}\right)$$

These values of energy and exergy are considered as the output of fan coils (*FC*) of the building; then the input energy and exergy are calculated as [32]:

(5) $$\phi_{FC,in}=\frac{\phi_{h}}{\eta_{e,FC}}$$

(6) $$\psi_{FC,in}=\frac{\psi_{h}}{\eta_{x,FC}}\cdot$$

The distribution pipes (*Dis*) have been considered as the inlet and outlet points representing the input and output energies. Therefore, the difference between input and the output values gives us the losses in pipes. Similarly, the exergy destruction is the difference between exergies at these two points [33]. The input energy and exergy are expressed as [34]:

(7) $$\phi_{Dis,in}=\frac{\phi_{FC,in}}{\eta_{x,Dis}}=\frac{\phi_{h}}{\eta_{x,Dis}\cdot \eta_{x,Fc}}$$

(8) $$\psi_{Dis,in}=\frac{\psi_{FC,in}}{\eta_{x,Dis}}=\frac{\psi_{h}}{\eta_{x,Dis}\cdot \eta_{x,Fc}}$$

The boiler (*B*) uses natural gas as fuel for heating water to be distributed in the building. Hence, the input of distribution can be considered the boiler output and the input values of both energy and exergy can be estimated as [35,36]:

(9) $$\phi_{B,in}=\frac{\phi_{Dis,in}}{\eta_{e,B}}=\frac{\phi_{h}}{\eta_{e,FC}\cdot \eta_{e,Dis}\cdot \eta_{e,B}}$$

(10) $$\psi_{B,in}=\frac{\psi_{Dis,in}}{\eta_{x,B}}=\frac{\psi_{h}}{\eta_{x,Fc}\cdot \eta_{x,Dis}\cdot \eta_{x,B}}$$

In addition to the heating load energy, energy used for lighting, ventilation, and domestic hot water (*DHW*) are also included in the heat-energy demand calculations. The components of the heating system need auxiliary electrical energy to be turned on. Hence, the energy demand (*ED*) for the first case is determined as:

(11) $$ED=\phi_{B,in}\cdot F_{P,B}+\left(P_{l}+P_{ve}+P_{aux,B}+P_{aux,Dis}+P_{aux,FC}\right)\cdot F_{P,elec.}+\phi_{DHW}\cdot F_{P,DHW}$$

and the total exergy demand (*XD*) becomes:

(12) $$XD_{}=\phi_{B,in}\cdot F_{P,B}\cdot F_{q,B}+\left(P_{l}+P_{ve}+P_{aux,B}+P_{aux,Dis}+P_{aux,FC}\right)\cdot F_{P,elec.}\cdot F_{q,elec.}+\phi_{DHW}\cdot F_{P,DHW}\cdot F_{q,DHW}$$

$F_{P},F_{P,DHW},F_{P,elec},F_{q},F_{q,DHW},\;and\;F_{q,elec}$ values are the energy ($F_{P}$) and exergy ($F_{q}$) factors of each sub-components of the building and are assumed to be equal to 1.1, 1.1, 3, 0.9, 0.9, and 1, respectively, as suggested by [8]. We assume $F_{P}=F_{P,DHW},\;and\;F_{q}=F_{q,DHW}$ because only one boiler produces the heating and domestic hot water.

For the 2nd case, the steps for the calculations are similar, with only difference being the re-calculation of the heating load, which equals 85% of the total heating load of the first case. This reduction in the heating load is because of the addition of the earth tube heat exchanger that cover 15% of the total heating load of the building. Based on the new value of heating load, the rest of the variables are calculated (please see the nomenclature for all symbols and the subscripts):

(13) $$\phi_{h-case2}=\left({\dot{Q}}_{trans}+{\dot{Q}}_{v}\right)\times \left(1-0.15\right)-\left({\dot{Q}}_{l}+{\dot{Q}}_{s}+{\dot{Q}}_{occ}+{\dot{Q}}_{app}\right)$$

The energy and exergy demand in 3rd case differ and were determined using the following expressions:

(14) $$ED=\phi_{B,in}\cdot F_{P}+\left(P_{l}+P_{ve}+P_{aux,B}+P_{aux,Dis}+P_{aux,FC}-P_{PV}\right)\cdot F_{P,elec.}+\phi_{DHW}\cdot F_{P,DHW}$$

(15) $$XD=\phi_{B,in}\cdot F_{P}\cdot F_{q}+\left(P_{l}+P_{ve}+P_{aux,B}+P_{aux,Dis}+P_{aux,FC}-P_{PV}\right)\cdot \;F_{P,elec.}\cdot F_{q,elec.}+\phi_{DHW}\cdot F_{P,DHW}\cdot F_{q,DHW}$$

The 4th case utilizes a trigeneration system instead of boiler, which works as a source for hot water, chilled water and electricity. In heating mode, the trigeneration system is used only for the hot water and electricity. Hence, the calculations are carried out only for the heating mode. The only energy and exergy need to be estimated before the trigeneration system is that for the natural gas energy, because only gas is used to produce energy. Therefore:

(16) $$ED=(\phi_{Tri,in})\cdot F_{P}$$

(17) $$XD=(\phi_{Tri,in})\cdot F_{P}\cdot F_{q}$$

We also consider *sustainability index* (SI), which provides a clear indication of the efficient use of resources [37]. Its value referred to the efficiency of the buildings or any device how they are efficient to use the resources energy. It can be calculated directly from the exergy efficiency as [15]:

(18) $$\eta_{x,building}=\frac{\psi_{h}}{XD}$$

(19) $$SI=\frac{1}{1-\eta_{x,building}}$$

Using these equations, the energy and exergy flows through the system components are estimated and taken as starting points for the next step, which is the exergoeconomic analysis. Several researchers used and/or illustrated the benefits of the ‘*specific exergy cost*’ (SPECO) method [5,38,39]. The SPECO approach allows the calculation of the loss of available work (exergy) based on the inefficiencies in each component of the system and its relation to the cost. Lazaretto and Tsatsoronis [40] demonstrated how to obtain definitions of exergy efficiencies using different forms of exergy, whether thermal, mechanical or chemical, and how to evaluate the costs associated with all the exergy streams entering and exiting a component of the system.

**Figure 1:**
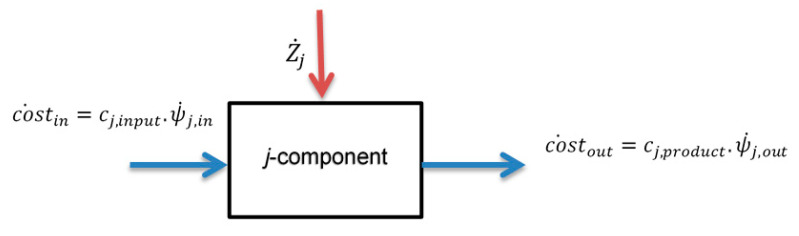
The schematic to show the basic concepts of the exergoeconomic analysis (*j*- component can be boiler, distributor, and fan coil).

The exergoeconomic balance equation for each component of the cycle can be written as (see Figure 1):

(20) $${\dot{cost}}_{in}+{\dot{Z}}_{j}={\dot{cost}}_{out}$$

where ${\dot{cost}}_{in}$ an ${\dot{cost}}_{out}$ represent the costs of energy of incoming and outgoing streams. $Z_{j}$ symbolizes the investment cost of the *j*-component.

Finally, the relation between the cost and the exergy of any component within the heating system can be determined based on the exergetic cost coefficient ($c_{j}$) which represents the cost per unit of exergy (USD/kWh), as shown in the following expressions:

(21) $${\dot{cost}}_{j,in}=c_{j,input}\cdot {\dot{\psi }}_{j,in}$$

(22) $${\dot{cost}}_{j,out}=c_{j,product}\cdot {\dot{\psi }}_{j,out}$$

Note that the exergetic cost coefficient at the exit of any component equals to the exergetic cost coefficient at the inlet of the next component.

By applying Equation (19) on the boiler or any component of the heating system (Figure 1, as adapted from [39], the cost of exergy output from each component of the building can be determined. The cost of the exergy, produced by the fan coils or Air Handling Unit (AHU), represents the cost of energy demand of the building. The price of natural gas is taken to be 0.0242 USD/kWh [38], whereas the electricity price is 0.072 USD/kWh [41]. (These cost values are taken in USD to be consistent with the predictions available in the literature).

**Figure 2:**
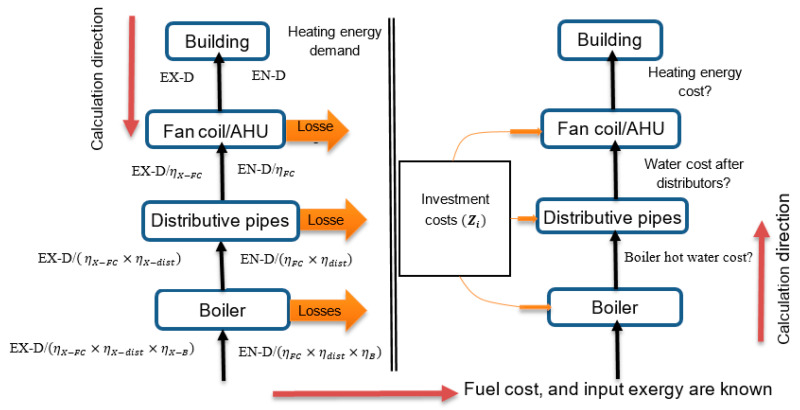
Schematic diagram explaining the calculation steps for energy, exergy, and cost analyses.

The enviroeconomic analysis has also been considered as the emission of unburned carbon CO_2_ has negative impact on the environment and increases the energy waste leading to climate change concerns. For this purpose, we can use an expression discussed in [18,42] that can be employed to estimate the amount of CO_2_ emission produced from any energy generating component as:

(23) $$Y_{CO2}=AN_{CO2}\cdot WY_{P}\cdot NH$$

where *Y*_CO2_ represents the CO_2_ emission in a year (kgCO_2_/year), *AN_CO2_* is the CO_2_ emission during fuel firing process (0.19 kgCO_2_/kWh) [38], *WY_P_* is the annual power (kWh) that was produced by using annual consumption (*Y_CO2_*) of fuel, and *NH* is the annular operating time in hours [42]. Furthermore, The emission for electricity is taken to be 0.38 kgCO_2_/kWh [43], and the international carbon price is considered to be 28 USD/tCO_2_ [44]. Figure 2 shows the main steps of calculations performed. Note that if carbon tax measures are widely adapted in the future, they can be added to the present analysis with a relatively simple approach. In that sense, the results here correspond to more conservative values (see World Bank Group 2019 [45] for recent research on carbon pricing).

## 3. Description of the Cases Considered

**Figure 3:**
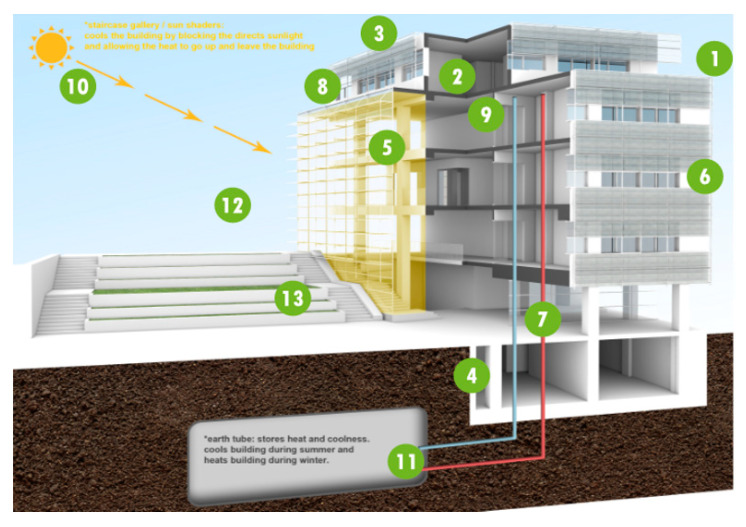
A schematic of SCOLA building at Ozyegin University campus, Istanbul, Turkey.

The School of Languages (SCOLA) Building was built in accordance with the master plan of the Ozyegin University campus situated in Cekmekoy district of Istanbul (north-west Turkey). The construction of the building was completed in 2014 with the partial support from the European Union 7th Frame Program New Energy Efficiency Demonstration for Buildings (NEED4B project. NEED4B was administered by CIRCE, Zaragoza, Spain and conducted in five different countries (Spain, Turkey, Sweden, Italy and Belgium). The Center for Energy, Environment and Economy (CEEE) was responsible from the Turkish demonstration building at Ozyegin University (as discussed in the final report; [46,47]). The SCOLA building was designed and constructed with the help from FIBA Holding, B-Design Architecture firm, both in Istanbul, and the University engineers and architects. Its energy use facilities were administered by the Energy Distribution Facility (EDF), resulting one of the most energy efficient buildings in Turkey (Figure 3) following the integrated engineering and architecture principles facilitated by CEEE.

More than 1450 students, 125 instructors and 10 administrative staff have been using SCOLA since September 2014. As the user capacity of the building is very high, there are three entrances to the building. The building consists of 66 classrooms for 20 occupants, four lecture rooms for 56 persons, two lecture rooms for 90 persons, three seminar rooms, 25 study rooms, 54 offices, cafeterias, baths, hallways and various service rooms. All classes are routinely used between 08:00 to 18:00 five days a week, accept summer months. All instructor offices are to be used between 08:00 to 17:00 according to schedule of instructors. The total floor area is 17,700 m^2^, whereas the net conditioned area is 15,093 m^2^. The building consists 6600 m^2^ of insulated external wall with an average U = 0.302 W/m^2^K, 3560 m^2^ of insulated roof with an average U = 0.248 W/m^2^K, 40% of which are underground, 1350 m^2^ of double glass aluminum-frame (with thermal break) windows (U = 1.6 W/m^2^K), 1500 m^2^ double glass curtain wall (U = 1.3 W/m^2^K), and 93 m^2^ external doors (U = 1.6 W/m^2^K). Moreover, it has an efficient external solar shade installed at specific sides of the building after a careful analysis of solar load [47]. The floors above the ground are ventilated naturally, however, a mechanical exhaust system is present too. The heating system used in the building involves the boiler, distributors, an air handling unit (earth tube, or ground air heat exchanger/pump system) used for ground floor of the building and fan coils for other floors. In addition, the building has an advanced management system that controls the lighting and conditioning leading to an essential reduction in energy demand. The necessary data required in estimating the energy and exergy efficiencies and the costs of all the components of the system have been continuously collected.

**Figure 4:**
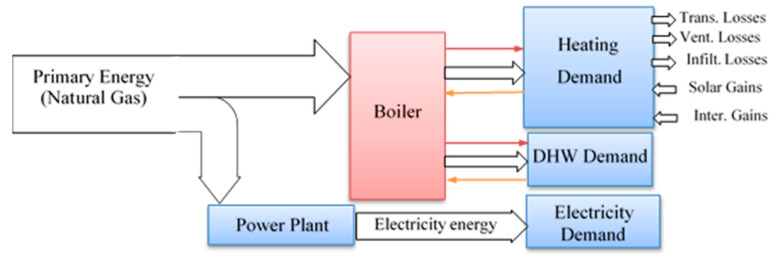
The building energy flow for the 1st Case.

The building has passed three stages of major improvements since it was built. These additions were decided by the stakeholders, i.e., by the University and the architectural firm. CEEE has influenced the process in line with the project objectives. In this paper we study only these three improvements, rather than many other energy efficiency measures considered for the building. The first was the addition of ground air heat exchanger (earth tubes). The second was addition of PV cells on the roof to exploit the solar energy and improving the building performance which plays an important role in such applications [26,27]. Addition of a tri-generation system to the building was the last improvement step. This system was installed at the Energy Distribution Center of the University, with partial contributions from the NEED4 project. Below, we provide the details of the energy, exergy, and the exergoeconomic analyses carried out to assess the importance of these three improvements on the building. As discussed before, in addition to the original case, we outline the results for three additions individually and all together, with a total of five different strategies considered in this paper:

*The 1st case*: This case represents the original concept which involves the architectural design of the building and its original heating system. The electricity is supplied by the national network, whereas the hot water is provided by the boiler (2200 kW) that uses natural gas as fuel. This case is considered the standard/baseline to compare against the other cases (see Figure 4).

**Figure 5:**
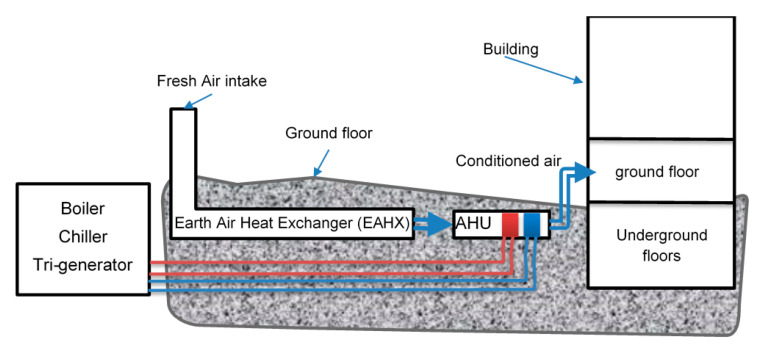
Schematic diagram of the Ground Air Heat Exchanger (GAHX) installation details and the corresponding air stream.

**Figure 6:**
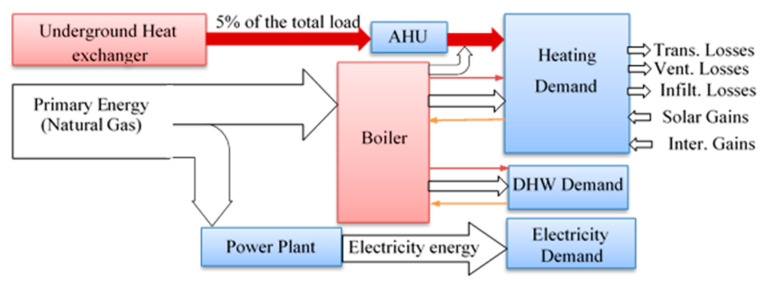
The building energy flow for the 2nd Case.

*The 2nd case*: Addition of the earth-tube (earth-air heat exchanger system) for partial cooling/heating to the building ventilation system corresponds to the 2nd case. The system, installed at the eastern side of the building, covers 1200 m² of land area, it is 10 m wide, at the depth of 2 m, with 72 m long horizontal pipes, and provides 10,000 m^3^/h air to the building. This heat exchanger is so far the largest installed system in Turkey. Figure 5 and Figure 6 explain its schematic diagram and how it is connected with the rest of heating system in the building [47].

*The 3rd case*: This case corresponds to the solar PV system installed on the building roof with a total capacity of 126 kWp. The system includes 504 Poly c-Si YL250P-26 PV modules (Yingli Solar, city, country) with 15.4% efficiency under standard test conditions (1000 W/m² incident radiation, 25 °C module surface temperature). These panels occupy 75% of the entire roof of the building. Produced DC electricity is converted by six 20 kWh string inverter units from Advanced Energy (formerly RefuSOL, city, country). All six inverters are combined into a distribution panel and connected to the grid through automated relay on floor electricity panel.

**Figure 7:**
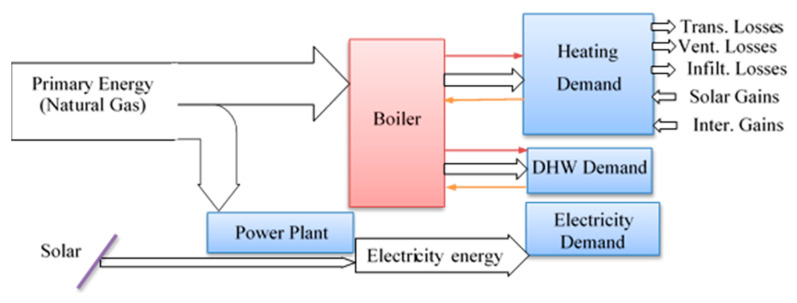
The building energy flow for the 3rd Case with PV panels installed on 75% of the building roof.

**Figure 8:**
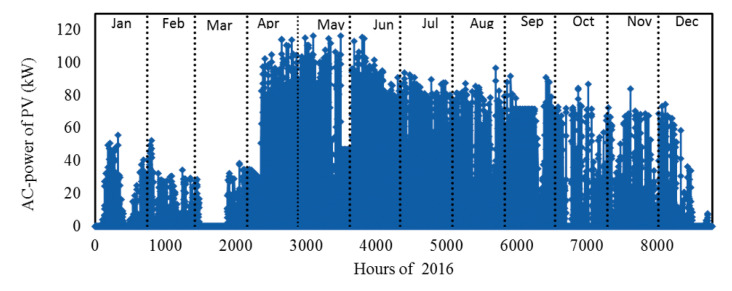
The actual AC power output of the PV system.

Figure 7 provides the schematic diagram of installed PV system; the details of this study was reported by by Sefer in his MS thesis and in the EU-FP7-NEED4B [47]. The AC-power output of the photovoltaic system is fed to the building. The hourly values of electricity generation for the entire year can be seen in Figure 8, as recorded by the Energy Distribution Center (EDC) at Ozyegin University, Istanbul. Note that due to system operation there has been some disruptions in the solar energy generation; here, we did use the data in our analysis as is, rather than normalizing it and assuming that it works as designed. Use of the real time data, with its own inherent imperfections make the current analysis more realistic.

**Figure 9:**
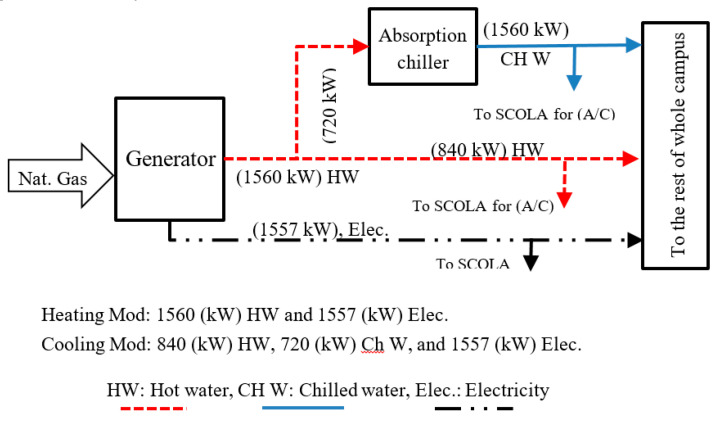
The tri-generator flow chart for heating and electricity production modes considered for the 4th case.

**Figure 10:**
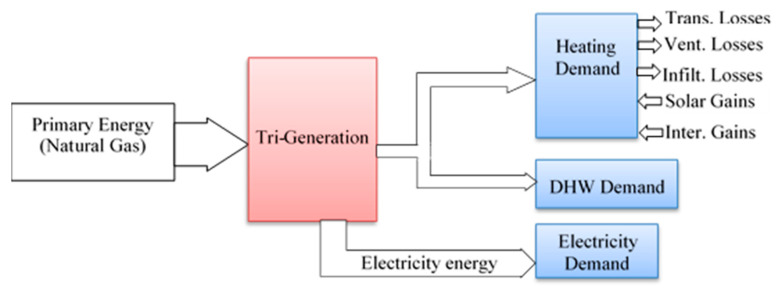
The building energy flow for the 4th case.

*The 4th case*: The most significant improvement for the building was achieved after adding a tri-generation system which simultaneously provides electricity and works as both a heating and cooling source. The tri-generator is utilized for the entire university campus, not only the SCOLA building considered in this study. Figure 9 indicates to the general mode of the tri-generator and how it provides f energy to the university campus. Here, we only consider the heating mode, so the tri-generator produces electricity and heat water. Figure 10 depicts the energy streams for the heating and electricity production modes. All the real-time data of the tri-generation system was provided by EDS of Ozyegin University.

*The 5th case*: For this final integrated case, the three additions; i.e., the air ground heat exchanger, the PV panels, and the tri-generation system are considered together. We note that the 5th case corresponds to the current operating conditions of the buildings with all alternative energy sources.

## 4. Results and Discussion

A pre-design tool ECBCS Annex 49 [10] is used to estimate the energy and exergy demand of the SCOLA building. Annex 49 is a task-shared international research project within the framework of the International Energy Agency (IEA) program on Energy Conservation in Building and Community Systems. Its aims are development, assessment and analysis methodologies that lead to reduce the exergy demand in the building and consequently the CO_2_ emission. Moreover, it focuses on the development of exergy distribution, generation and storage system concept [6,10,11].

The energy and exergy efficiencies of the heating system were calculated based on energy and exergy balances of each component dynamically for five cases. The economic aspects of the operations were considered by applying the exergoeconomic analysis using a dynamical methodology [30]. In addition, environmental impact is considered to determine the cost of generated emission due to energy consumption. Lastly, a detailed comparison between these five cases was provided; all the corresponding data are listed in the Appendix A.

**Figure 11:**
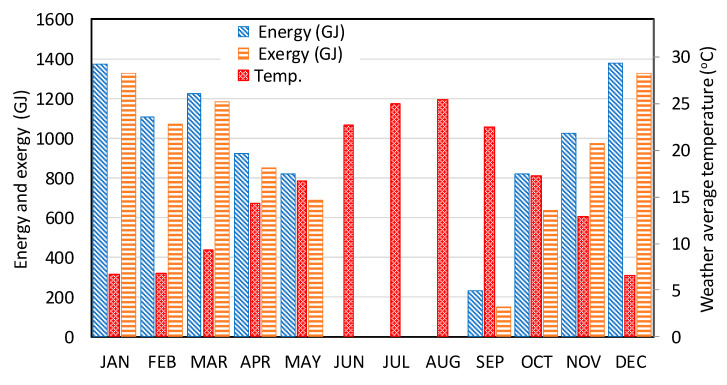
The monthly energy and exergy demands (GJ) and ambient temperature (°C).

The monthly energy and exergy demand and the weather temperature data used for this study are shown in Figure 11. It is clear that the energy demand changes in line with the change of ambient temperature. Note that there is no heating demand in the months of June, July and August. In addition, the ambient temperatures in some days of May and September are higher than 22 °C; therefore, the demands in these two months are small relative to others. The maximum load occurs in December and January, respectively. The monthly exergy demand is also plotted in this figure (see the Appendix A for the data). Note that, in this study we only focus on heating and electricity demands, and cooling demand is not considered. More details about the monthly loads and monthly energy of all types for all case are listed in the Appendix A, Table A1.

**Figure 12:**
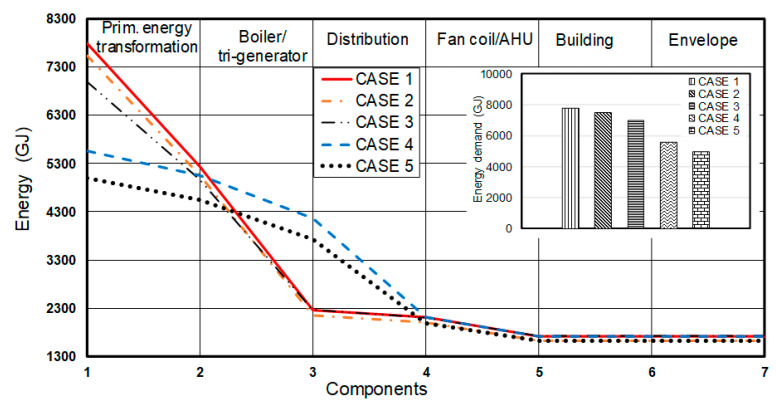
The annual energy flow from the source to the building envelope.

Figure 12 shows the dynamically calculated annual energy flow through the building and its heating system for all five cases. It can be observed that the highest energy demand is for the 1st case which depends only on the architectural design and the initial heating system. The importance of reducing the heating load of the building can be seen for the 2nd case, which corresponds to the addition of the Ground Air Heat Exchanger. However, this reduction is relatively small, 4% relative to the 1st case. A higher reduction in energy demand, about 10%, is observed for the 3rd case. The impact of the tri-generation system is much larger, results in almost 29% reduction in annual energy demand. Integrating all the improvement processes decreases the energy demand by 36%, which corresponds to the 5th case. This level of improvement in building performance has the potential to lead to significant reduction in energy demand not only in the campus, but all over the world.

**Figure 13:**
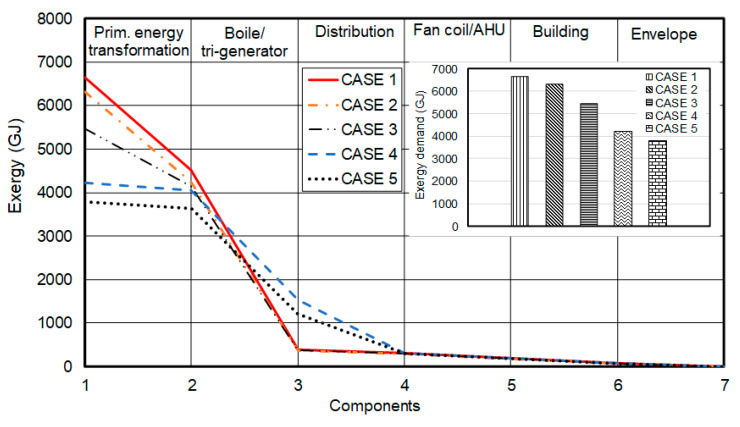
The annual exergy flow from the source to the building envelope.

In addition to energy flow, annual exergy flows were also dynamically estimated for all cases considered for the building and its heating system at each stage. The exergy demands reductions for the 2nd and 3rd cases, relative to the 1st case, are 5% and 18%, respectively. It can be noticed that the exergy reduction is mostly due to the PV-panels (the 3rd case) which contributed significantly to the enhanced performance of a building. The 4th case helped reducing the exergy demand around 36% relative to the 1st case. The integrated 5th case is, as expected, to provide the maximum reduction in exergy consumption, by 43%. Figure 13 clearly shows the impact of the use of a trigeneration system for the reduction of the building energy demand, and consequently the CO_2_ emission.

**Figure 14:**
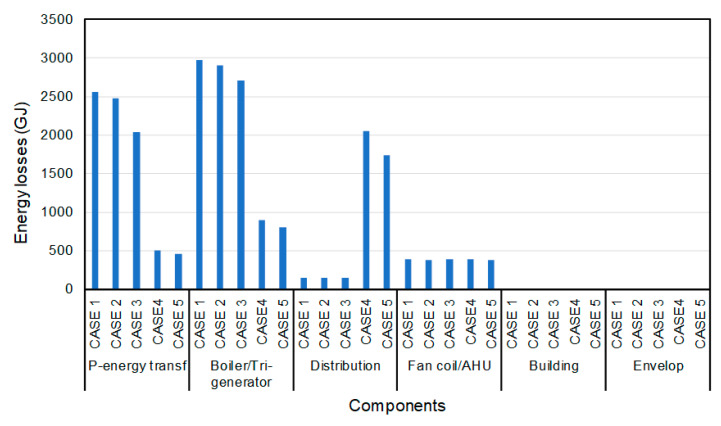
The annual energy losses of sub-components for all cases.

**Figure 15:**
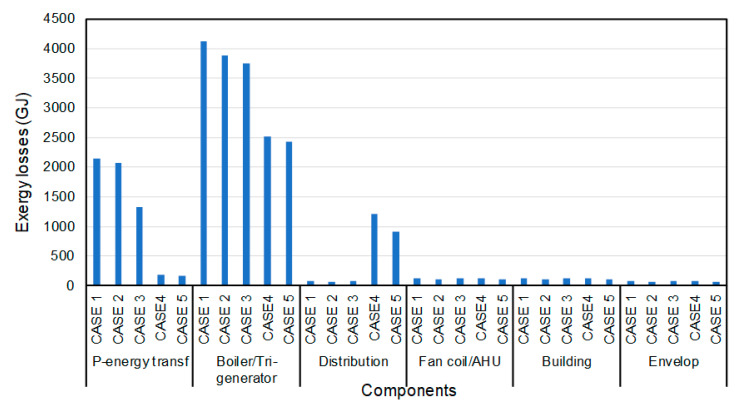
The annual exergy losses of components for all cases.

It is also important to outline the annual energy and exergy losses for system sub-components, as shown in Figure 14 and Figure 15. This concept was also discussed by Schmidt [8,10]. The smallest losses are observed for the 5th case for both the primary energy transformation and the boiler/tri-generation stage sub-components. For the 4th and 5th cases, the distribution pipes have the largest values of losses with respect to other cases. For the primary energy transformation stage, the energy source is natural gas. Its energy and exergy factors (*F* factors; see Equations (11) and (12)) are 1.1 and 0.9, respectively, while these factors for the electricity are 3 and 1, respectively. Hence this large difference can be seen in the results presented. For the first three cases (1st, 2nd and 3rd), the input energy is based on electricity and natural gas, and output is only for hot water. For the last two cases (4th and 5th case) the input energy is obtained only from natural gas. The corresponding energy, therefore, is lower than that for the first three cases. The output is the sum of electricity and hot water, which is higher than the output energies of the first three cases. For the distribution stage, in the first three cases, both the input and output energy are due to hot water; therefore, the losses are only because the reduction in water temperature within the pipes. In the last two cases the losses of this stage are higher, because that the electricity and hot water represent the input power of this stage while the output is only the hot water energy. In fan-coil/AHU component, the losses of the 2nd case are less than the corresponding value for the 4th case, as the heating load is the less.

Exergy losses (or destructions) do not differ from the energy losses, as shown in Figure 15. The original 1st case has higher losses than 2nd, 3rd and 4th cases; again, as expected, the smallest losses are observed for the 5th case. The highest losses of all cases occur at the generation stage (boiler of the tri-generator), whereas the second largest loss occurs due to primary energy transformation. We note that the losses of the first three cases are higher than those of the last two. Similar to the energy losses, the distribution stage has the highest exergy losses.

**Figure 16:**
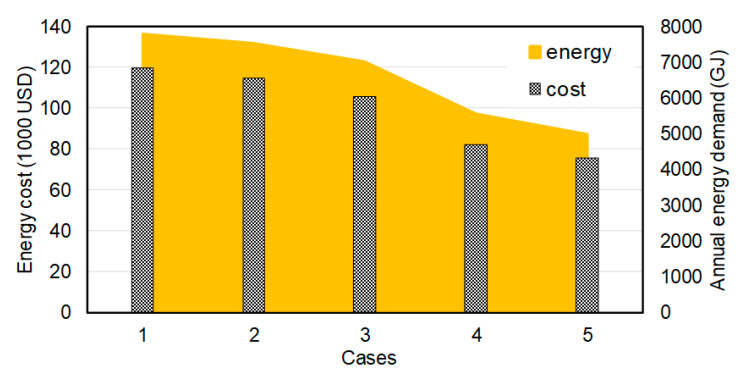
The annual energy demands and their costs for all cases.

In addition to the energy and exergy flows and losses, the energy consumption cost is quantified for all five cases using a dynamic exergoeconomic analysis [30]. The costs of energy consumption of all the cases are presented in Figure 16. The figure gives an overview of the importance of alternative energy use. For the 2nd case, 4% reduction in annual energy demand was achieved using the earth tube which corresponds to 4740 USD savings annually. This reduction is larger for the 3rd case, i.e., the use of PV panels to provide electricity to the building. With the help of PV panels, the energy demand was reduced around 12% with 13,890 USD annual saving. On the other hand, the 4th case, the use of a trigeneration system, results to a reduction of 31%, corresponding to 37,244 USD savings annually. Moreover, 37% reduction in annual energy demand can be reached when engineering applications used for the 2nd, 3rd and the 4th cases are integrated, as in the 5th case.

**Figure 17:**
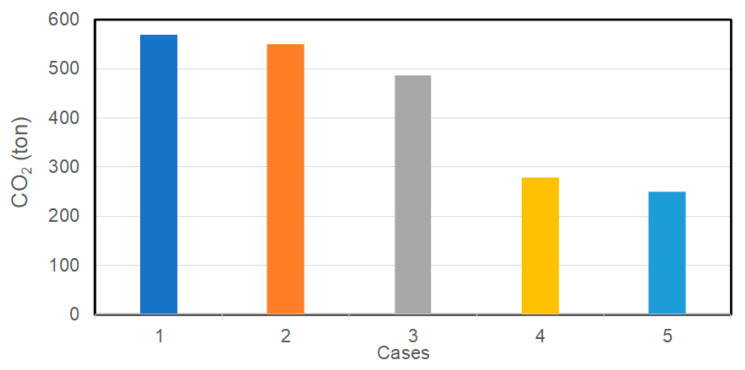
The annual CO_2_ emission (ton/yr) for five cases considered.

The environmental considerations of the system are outlined in Figure 17. The 2nd and 3rd cases reduce the CO_2_ emissions by 4% and 15%, respectively. A 51% reduction can be achieved by using a trigeneration system, as shown for the 4th case. The overall system improvements corresponding to the 5th case result in 56% reduction in CO_2_ emissions. This improvement is significant and shows that the trigeneration system should definitely be considered for financial investment by the stakeholders.

The exergies corresponding to all five cases and the building components are listed in Table 1. Using these values, the exergy efficiencies and sustainability indexes of all components of a system can be estimated. These two values indicate how efficient the energy demand is used by the building and its heating system. For instance, the exergy efficiency and sustainability index of the boiler for the 1st and 2nd cases are 5.7% and 1.06, respectively, whereas for the 3rd case they are 7% and 1.07, respectively. For the 4th and 5th cases a tri-generation system used rather than simply a boiler. The exergy efficiencies and sustainability indexes for these two cases are (36%, 1.56) and (32%, 1.47), respectively. These results highlight the impact of the use of a tri-generation system and how positively it affects the building energy performance.

**Figure 18:**
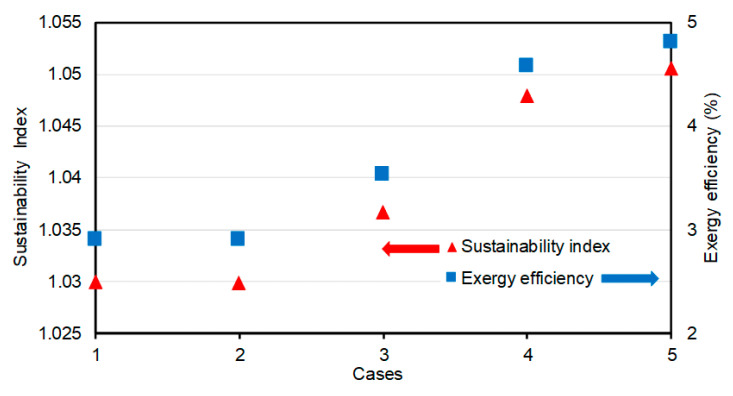
The exergy efficiency and sustainability index for each case considered.

Figure 18 depicts the exergy efficiency and sustainability index values of the building for each of the five cases considered. For the 1st (original design) case, the exergy efficiency and sustainability index are 3% and 1.03, respectively. The 2nd case values are identical with their values for the original design. The total exergy demand and the exergy load of the building are reduced, but even the exergy efficiency and sustainability index for this case are still identical with those for the baseline (original design) case. The sustainability indexes for the 3rd, 4th, 5th cases are 1.036, 1.048, and 1.051, respectively, whereas the corresponding exergy efficiencies are 3.5, 4.6, and 4.8, respectively. The higher values of these indexes indicate that all of the new scenarios enhance the performance of the building, although with varying degrees.

## 5. Conclusions

In this paper we have presented a detailed performance analyses of an existing high-performance building with five scenarios based on different alternative energy sources, including the original design, three different additions of energy systems (earth-tube heat exchanger, roof-top PV panels, or trigeneration unit), and the integrated design including all three systems. The building analyzed is an academic building (SCOLA) in the campus of Ozyegin University in Istanbul, Turkey. For the five cases considered the energy and exergy flows, the components losses, the energy costs, and CO_2_ emission were calculated dynamically for the entire heating season; the corresponding data are given in the Appendix A. Furthermore, the exergy efficiency and sustainability index of the building for all cases were determined. The results obtained can be summarized as follows:

(1) The consideration of the 2nd, 3rd, 4th, and 5th cases show the potential reduction the total energy demand is around 3%, 10%, 29%, and 36%, respectively, relative to the original design. These additional considerations correspond to 4%, 12%, 31%, and 37% reduction in annual cost of energy for each case, in respective order.
(2) The annual exergy demand can be reduced by 5%, 18%, 36%, and 43%, respectively, following the four scenarios considered beyond the original design. These values show the potential broader impact of each improvement considered.
(3) The CO_2_ emission can be lowered by 3%, 15%, 51%, and 56% relative to the original design by implementing the 2nd, 3rd, 4th, and 5th scenarios, respectively.
(4) The exergy efficiency and sustainability indexes for the case based on using tri-generation system (the fourth case) are 36% and 1.56, respectively. These values are quite high compared to those for the original case (6% and 1.06, respectively).
(5) Maximum enhancement in building performance can be achieved by using a tri-generation system; the second highest impact comes from the roof-top PV panels.
(6) The maximum improvements in exergy efficiency and sustainability index of the building can be achieved by considering the 4th (trigeneration) and the 5th (comprehensive) cases.
(7) The dynamic analysis presented provides a clear picture of buildings performance and should be preferred over simpler static analysis.

## Figures and Tables

**Table 1:** The exergy values (in GJ) of the building components for all five scenarios.

Components	Case 1	Case 2	Case 3	Case 4	Case 5
Input	6648	6324	5471	4226	3792
After primary energy transformation	3434	3209	3330	4044	3628
After (boiler/trigeneration)	382	363	382	1525	1205
After distribution	311	295	311	311	293
After fan coil/AHU	194	184	194	194	183
After room	75	71	75	75	70
After envelope	0	0	0	0	0

## Appendix A

The table below provides the details required for the cascaded analyses presented in the paper. All these data are for the calendar year of 2016 and based on the actual measurements for SCOLA Building, as obtained for from the Energy Distribution Center (EDC) at Ozyegin University, Cekmekoy, Istanbul, Turkey.

The monthly ambient average temperatures (second column), total energy demand (third column), heating loads (fourth column), and energy production from the different alternative energy systems are listed (the fifth and sixth columns) corresponding to the 2nd, and 3rd cases, respectively. The 2nd and 3rd cases show for the contributions of two different renewable energy modalities to the 1st case. The energy demand of the 4th case, which includes the contributions from the trigeneration system, are listed in the seventh column. The last column shows the reduced energy load after integrating all three systems together. Note that there is no heating demand for June, July and August months, because the average temperatures of these months are higher than 22 °C. Moreover, the loads of May and September are small comparing to other months, because the average temperatures in some days of these two months are higher than 22 °C.

**Table A1:** The monthly outline of data used in the analyses presented in the paper.

Months (2017)	Average Temp. (°C)	Total Energy Demand (GJ) The 1st Case	Heating Load (GJ)	Earth Tube Systen (GJ) The 2nd Case	Solar Energy System (GJ) The 3rd Case	Tri-Generation System (GJ) The 4th Case	All Energy Systems Together (GJ) The 5th Case
Jan	7	1200	264	41	21	856	450
Feb	7	971	214	33	24	692	387
Mar	9	1071	236	37	17	764	396
Apr	14	809	178	28	95	577	560
May	17	719	158	25	100	513	548
Jun	23	0	0	0	101	0	321
Jul	25	0	0	0	89	0	281
Aug	25	0	0	0	83	0	265
Sep	22	202	44	7	81	144	323
Oct	17	719	158	25	70	513	452
Nov	13	898	198	31	66	641	496
